# Development of a Controlled Continuous Low-Dose Feeding Process

**DOI:** 10.1208/s12249-021-02104-9

**Published:** 2021-10-12

**Authors:** Sara Fathollahi, Julia Kruisz, Stephan Sacher, Jakob Rehrl, M. Sebastian Escotet-Espinoza, James DiNunzio, Benjamin J. Glasser, Johannes G. Khinast

**Affiliations:** 1 Research Center Pharmaceutical Engineering (RCPE) GmbH, 8010 Graz, Austria; 2 Graz University of Technology, Institute of Process and Particle Engineering, 8010 Graz, Austria; 3 Oral Formulation Sciences and Technology, Merck & Co., Inc., Rahway, New Jersey USA; 4 Department of Chemical and Biochemical Engineering, Rutgers University, Piscataway, New Jersey 08854 USA

**Keywords:** Loss-in-weight feeder, Low dose feeding, Continuous feeding, Iterative learning control, Feed forward control

## Abstract

This paper proposes a feed rate control strategy for a novel volumetric micro-feeder, which can accomplish low-dose feeding of pharmaceutical raw materials with significantly different powder properties. The developed feed-forward control strategy enables a constant feed rate with a minimum deviation from the set-point, even for materials that are typically difficult to accurately feed (e.g., due to high cohesion or low density) using conventional continuous feeders. Density variations observed during the feeding process were characterized via a displacement feed factor profile for each powder. The characterized effective displacement density profile was applied in the micro-feeder system to proactively control the feed rate by manipulating the powder displacement rate (i.e., computing the feed rate from the powder displacement rate). Based on the displacement feed factor profile, the feed rate can be predicted during the feeding process and at any feed rate set-point. Three pharmaceutically relevant materials were used for the micro-feeder evaluation: di-calcium phosphate (large-particle system, high density), croscarmellose sodium (small-particle system, medium density), and barium sulfate (very small-particle <10 μm, high density). A significant improvement in the feeding performance was achieved for all investigated materials. The feed rate deviation from the set-point and its relative standard deviation were minimal compared to operations without the control strategy.

## INTRODUCTION

Tablets and capsules are the most common forms of drug products (1) and comprise in total more than 70% of oral dosage forms (2). Many factors, such as the variability in raw material’s physical properties (e.g., bulk properties) and manufacturing process disturbances (3), can affect the quality of final drug products. The drug product’s quality and consistency are assured through well-designed development and manufacturing process within the Quality by Design (QbD) framework (4). Over the last 10 years, continuous manufacturing has been increasingly applied in the pharmaceutical industry due to its many potential benefits (5, 6). Continuous powder feeding is a common unit operation for all continuous manufacturing (CM) processes for both active pharmaceutical ingredients (APIs) and excipients (7). Powder feeders play an important role in the CM process: *they maintain the steady state of the process and deliver the pharmaceutical ingredients to the downstream process*(6, 8), e.g., continuous granulation, tableting, and coating. Individual feeders continuously deliver the APIs and excipients according to the formulation and at pre-defined feed rates (6). Consistent feeding of materials requires a good understanding of the material properties and the manufacturing process. Additionally, an automated process control system is essential to address both the measurable and the non-measurable process disturbances in real-time(3). Control of the feeding operation is a primary component of a system’s control strategy since the input of the continuous process directly affects the output, and thus, the critical quality attributes of a drug product, such as assay and content uniformity (6).

Loss-in-weight (LIW) feeders are frequently used in the pharmaceutical CM process to maintain consistent feeding into subsequent unit operations. The principle of LIW feeding involves the constant monitoring of the mass (i.e., weight) of material in the feeder while discharging and constantly adjusting the rate of discharging to maintain a mass flow rate (9). The mass of the feeder is monitored via a balance under the feeding unit. LIW feeding stands in contrast to gain-in-weight(GIW) because in the latter the balance is placed outside of the feeding unit to collect the material discharged out of the feeder. From a processing perspective, the use of LIW feeders can be used in conjunction with other unit operations while the GIW feeders need to terminate into the balance in which the material mass is being measured.

The feeding range of feeders depends on the feeder’s size and tooling (e.g., screws and screens) and the material properties (9). Although the use of feeders is well established in many industries and has been successfully applied in various processes, there are limitations as to the specific types of materials and the minimum feasible feed rate. Especially at low feed rates (< 1 kg/h), LIW feeding is challenging due to feed-rate fluctuations associated with the screw conveying principle, problems during the hopper refilling, and associated feed-rate variations due to changes in the powder bed height. Moreover, bridging and adhesion of cohesive materials are associated with the screw conveying method, leading potentially to blockage of the feed channel (10–13). In continuous processes, blending elements are typically designed to reduce the variability caused by the feeding operation (6). However, some studies (14, 15) indicate that the variability and disturbance during the feeding operation can affect the performance of downstream unit operations and the final product quality. To control the feeding performance of LIW feeders, the feeder tooling selection was matched to the material properties (9, 12, 13, 16, 17). In addition, individual feeder control strategies have been developed to reduce the variability of the fed material’s concentration (6, 18).

To control the feed rate, feeders have an integrated balance to inform a closed-loop controller of the actual discharged mass and then adjust the feed rate accordingly by speeding up or slowing down the discharging element (e.g., screws or paddles). The closed-loop control can be, e.g., proportional integral (PI) or proportional integral derivative (PID)(11). Such a feed-back control strategy allows one to monitor the plant’s output (i.e., the feed rate computed based on the balance raw signal) and take actions (e.g., adjusting the screw speed) in order to attenuate the effects of any disturbance in the feed rate. A feed-forward control strategy makes it possible to take actions based on the process knowledge and the measured disturbances (a feed-forward signal) before these disturbances affect the plant’s output (3). However, it is impossible to monitor the feed rate in real-time and actively take action if deviations from the feed-forward signal (unmeasured disturbances) occur. Especially with regard to pharmaceutical products, it is important to monitor the process in-line, detect possible disturbances (deviations from the feed-forward signal), and take actions before the process disturbances (indicated by the feed-forward signal) affect the final product quality. For example, in the tableting environment, this can affect the content uniformity, weight, and functionality of the final tablets (19).

A strategy combining feed-back and feed-forward control is required to suppress predictable (measured) disturbances proactively and to monitor the process for possible unmeasured disturbances in real-time(3). Some studies investigated control at a system level, using feed-back control strategies (20–23) and feed-forward control models (24, 25) to control the manufacturing processes. Other authors (3, 26, 27) proposed a combined feed-forward feed-back control system for continuous manufacturing process. Furthermore, there are studies on the application of iterative learning control for weighing the powder materials (28, 29).

Low-dose feeding of cohesive materials, such as highly potent APIs (HPAPIs) and lubricants (8), is a challenge due to the inherent feeder variability (11). In our previous study (30), a novel micro-feeder system was introduced, which enables the feeding of powders with diverse powder properties (e.g., size, density, flow properties, and cohesivity) at feed rates as low as 1 g/h. In addition, one API and one spray-dried intermediate (SDI), both highly cohesive, were fed to highlight the industrial applicability of the micro-feeder system. Based on the volumetric feeding principle, this micro-feeder system yields a constant volume of powder per unit time. In the absence of a control strategy, a consistent feed rate is determined by the constant powder mass distribution in the feeder cartridge. Depending on the formulation, even slight deviations in density and feed rate may lead to an out-of-specification event during continuous manufacturing of low-dose drug products. To address this issue, in this follow-up study, a strategy for controlling the feed rate during the feeding process was developed and evaluated by feeding di-calcium phosphate, croscarmellose sodium, and barium sulfate.

In general, the micro-feeder enables a continuous supply of low-dose materials. This is highly relevant for continuous manufacturing routes, where no pre-blending is desired and raw materials can be fed separately. The applicability of the micro-feeder in continuous operation mode was for example shown in a hot-melt extrusion process (31), in which variation of content uniformity was in the same range as with pre-blend preparation.

## MATERIALS AND METHODS

### Materials

Di-calcium phosphate (dibasic calcium phosphate, Sigma-Aldrich, UK), croscarmellose sodium (sodium carboxymethylcellulose, Sigma-Aldrich, UK), and barium sulfate (Sigma-Aldrich, UK) were used in this work. They were selected to demonstrate the feeding variability relative to the various powder properties.

### Powder Characterization Techniques

#### Particle Size Distribution Measurement

Particle size distributions (PSDs) of the materials were measured via laser light diffraction techniques (Helos/KR, OASIS/L dry dispersing system Sympatec, Clausthal-Zellerfeld, Germany). A vibrating chute was used to transport the powder in a controlled way to the dispersing unit. A dispersion pressure of 2.5 bar was applied.

#### Bulk and Tapped Density Measurements

The bulk (poured) and tapped densities (BD and TD) of materials and mixtures were analyzed via a Pharmatest PT-TD200, a standardized method described in the United States Pharmacopeia (32). The bulk density (BD, g/cm^3^) is determined by pouring powder carefully into a standard 250 mL cylinder. For tapped density (TD, g/cm^3^), the powder in the cylinder is mechanically tapped, and then, the volume of the powder is recorded. Bulk and tapped density are calculated by dividing the powder mass by volume.

### Methods

#### Experimental Setup

**Fig. 1:**
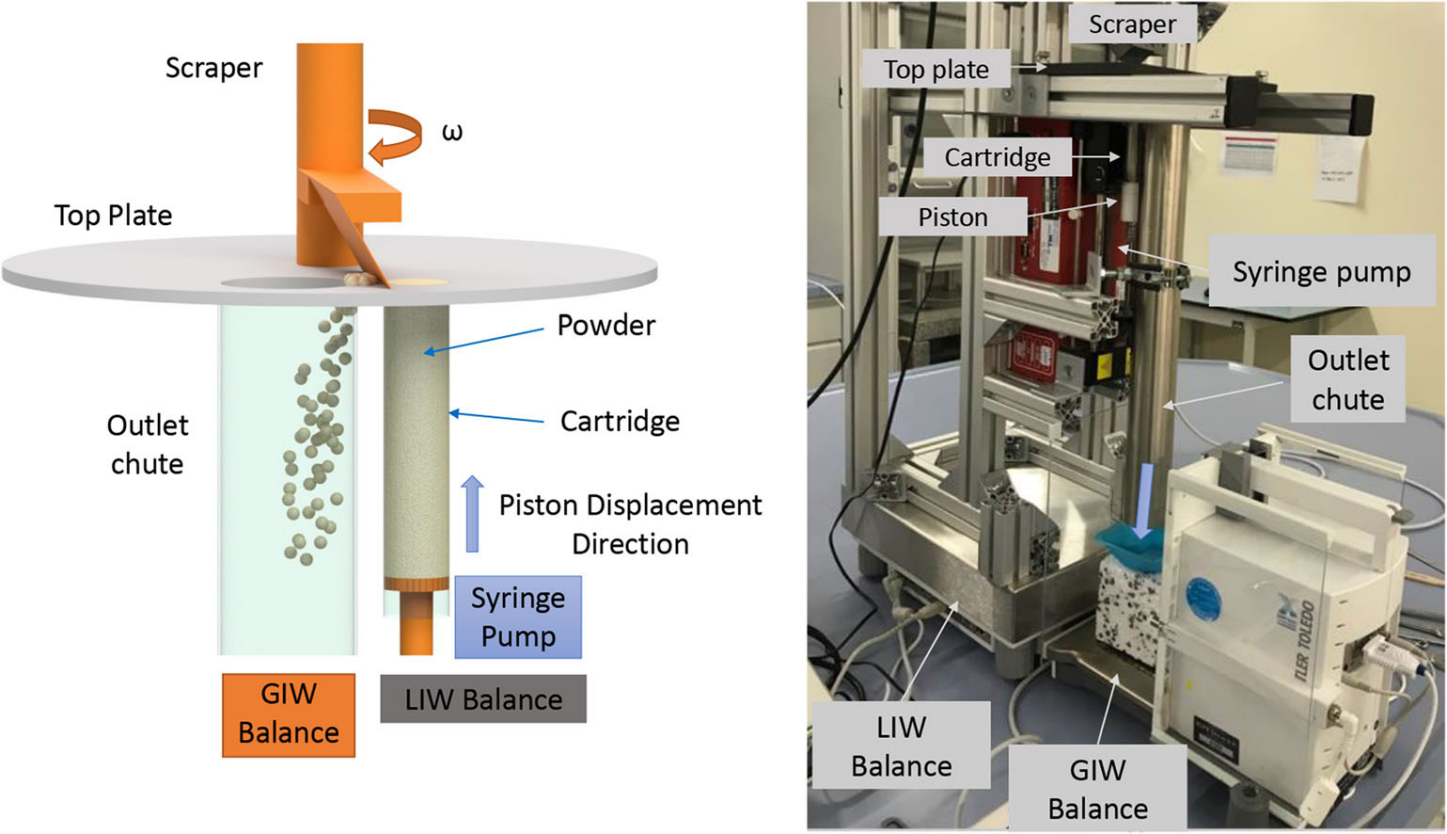
Micro-feeder system: schematic of the system (left), experimental setup (right). LIW, loss in weight; GIW, gain in weight

The micro-feeder system, described by Fathollahi et al. (30), was augmented to include a LIW option. The micro-feeder consists of a cartridge, which contains the powder, a moveable piston actuated via a syringe pump to displace the powder, and a scraper to transport the material to the process. It is combined with a weighing balance (Mettler Toledo, XPE204, 0–220 g with readability of 0.0001 g) at the outlet that measures the feeder’s output rates by recording the accumulated mass at the outlet of the feeder (gain in weight (GIW)). Additionally, the micro-feeder is placed on another balance (Mettler Toledo, XSR32001L, 0.1–32100 g with a readability of 0.1 g) to monitor the weight loss over time (LIW). A schematic of the micro-feeder system is shown in Fig. 1.

#### Data Acquisition and Equipment Integration

**Fig. 2:**
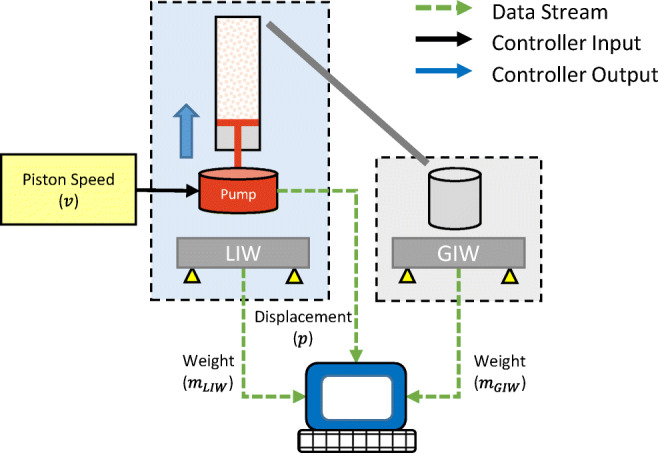
Schematic of the set up for the calibration runs

The piston is driven by a syringe pump (NE-1000 programmable single syringe pump, New Era instruments via New Era pump systems, USA), which can be connected to a PC via a serial port (RS232). The dosed volume can be read from the serial connection. Furthermore, it is possible to write a new displacement speed set-point and start/stop the pump via the serial port. Both balances are also connected via the serial interface. They provide the actual weight value that is differentiated and filtered in a post-processing step (see Fig. 2). All available parameters are acquired at a sampling frequency of 1 Hz using Matlab® (Mathworks, Natick USA). At each time point, the data acquired from the syringe pump and the balances are recorded, and the piston position is computed (see Eq. (1)).

1 $$ p={V}_{in}/{A}_{cart}, $$

where *p* is the calculated position of the piston, *V*_*in*_ is the dosed volume (which is read from the pump via the RS232 interface), and *A*_*cart*_ is the cross-sectional area of the cartridge. The set-point for displacement speed *v* is written to a text file, including a timestamp to allow time-aligned data processing.

#### Data Processing

All data processing discussed in this work was performed using Matlab® (Mathworks, Natick USA). The feed rate was obtained via a Savitzky-Golay derivative filter (33, 34) with window lengths of 2 min for the GIW data and 10 min for the LIW data, both using a second-order polynomial.

#### Micro-feeder Characterization Methodology

The feed rate in the micro-feeder system can be adjusted through the piston’s displacement speed. The micro-feeder system assumes the piston displaces the cartridge volume continuously and at the specified rate (i.e., instantaneous adjustment). This ensures a constant mass feed rate, provided that the density in the cartridge is constant initially and during feeding due to pre-conditioning. The pre-conditioning procedure is designed as an essential step prior to the feeding process to eliminate cavities and inter-particle voids. During this step, the powder in the cartridge is tapped and compacted to the powder tapped-density state. Details of the pre-conditioning procedure are provided in our previous work (30).

In this study, the scraper rotating speed was set to 10 rpm. The high-precision balance was used as a catch (gain-in-weight) balance (i.e., GIW data) at the outlet of the micro-feeder to measure the accumulated mass of the material fed. At the same time, the other balance, on which the micro-feeder is located, recorded the mass loss (i.e., LIW data) of the micro-feeder.

Volumetric displacement *ΔV* of the powder on top of the cartridge during time interval Δ*t* is not known precisely. However, it can be approximated via the volumetric piston displacement at the bottom of the cartridge (*ΔV* ≈ *v* · *A*_*cart*_ · Δ*t*), where *v* is the constant piston displacement speed in the cartridge and *A*_*cart*_ is the cross-sectional area of the cartridge. Accumulation of mass at the catch balance (relating to the powder exiting at the top, *Δm*) was measured. We defined a property termed “the effective displacement density,” *ρ*_*ED*_ as $\frac{\varDelta m}{\varDelta V\ }$, which is an approximation of the actual bulk density of the powder at the exit*.* The feed rates are determined based on the generated data each second (${\dot{m}}_{\mathrm{f}}=\varDelta m/\varDelta t$). Since the micro-feeder system is based on the volumetric principle, a variation in the effective displacement density along the cartridge causes the feed rate variation over the process time. The effective displacement density is plotted as a function of displacement for each material and is termed a “displacement feed factor.” The displacement feed factor profile represents the uncontrolled feed rate profile for each material.

A benefit of the micro-feeder system is that the system is robust and stable, and feeding is reproducible. Specifically, the displacement feed factor is reproducible yet unique (30) for each material and depends on the powder properties, such as the particle size, elastic behaviour, and bulk and tapped densities. Most importantly, the displacement feed factor is not affected by the feed rate (30) for the tested materials in the investigated ranges. This property makes it possible to apply the displacement feed factor in a feed-forward control strategy. The piston displacement speed is calculated from the actual piston position to compensate for the displacement feed factor profile and achieve a feed rate closer to the set-point over the entire length of the cartridge.

#### Calibration Runs

Calibration runs were performed prior to controlled feeding to determine the displacement feed factor for each material. A schematic of the setup of the calibration runs is shown in Fig. 2. The piston displacement speed (Eq. (2)) is calculated based on the powder mass (M) in the cartridge length (L) considering the desired feed rate (${\dot{m}}_{set}$),

2 $$ Piston\ speed,v\ \left[\frac{mm}{\mathit{\min}}\right]=\frac{{\dot{m}}_{set}\ \left[\frac{g}{\mathit{\min}}\right]}{\frac{M}{L}\ \left[\frac{g}{mm}\right]} $$

The initial powder mass is assumed to be constantly distributed along the cartridge and to remain constant during the feeding process. Therefore, the piston displacement speed is set to a fixed value for the entire run.

The calibration runs were performed at feed rates of 5 g/h and 10 g/h and at the lowest possible piston displacement speed of 0.1 mm/min, which is the limit for the syringe pump. The data from the GIW balance were used to calculate the displacement feed factor profile of each material.

#### Control Strategy

The feed rate in the micro-feeder system was determined based on the displacement speed of the piston in the cartridge and the displacement feed factor, i.e., the piston displacement speed acted as a manipulated variable. The control concept consisted of two stages: (1)feed-forwardcontrol and (2) iterative learning control.

#### Feed-Forward Control

Actual piston displacement information and the material-specific feeding behaviour were used to control the feed rate in the feed-forward mode. The piston position data were obtained from the syringe pump. The material-specific feeding behaviour was represented by the displacement feed factor, which was determined in the calibration runs. A polynomial function describing the displacement feed factor over the displacement based on the calibration results was defined for each material and used to calculate the piston displacement speed required for achieving the desired feed rate. The polynomial function is given in Eq. (3) with the displacement *p* and the polynomial coefficients *α*:

3 $$ {\rho}_{poly}(p)=\sum \limits_{i=0}^8{\alpha}_i{p}^i $$

The high-order polynomial was chosen in order to capture the more complex shape of the displacement feed factor curve of di-calcium phosphate (30).

For the feed-forward control, the syringe pump was run at the calculated piston displacement speed, which was adapted according to the piston displacement. Nominal feed rate ${\dot{m}}_{nom}$ for feed-forward control in Eq.(4) can be expressed as a function of the effective displacement density (modelled by the polynomial in Eq. (3)) in the calibration run in combination with piston displacement speed *v* and cartridge cross-sectional area *A*_*cart*_,

4 $$ {\dot{m}}_{nom}={\rho}_{poly}(p)\cdot v\cdot {A}_{cart} $$

By rearranging Eq. (4), the required piston displacement speed can be calculated. The feed-forward control is based on the idea that the nominal feed rate is identical to the feed rate set-point${\dot{m}}_{ref}$, i.e., ${\dot{m}}_{nom}={\dot{m}}_{ref}$. Consequently, the piston speed is computed by

5 $$ v=\frac{{\dot{m}}_{ref}}{\rho_{poly}(p)\cdot {A}_{cart}} $$

**Fig. 3:**
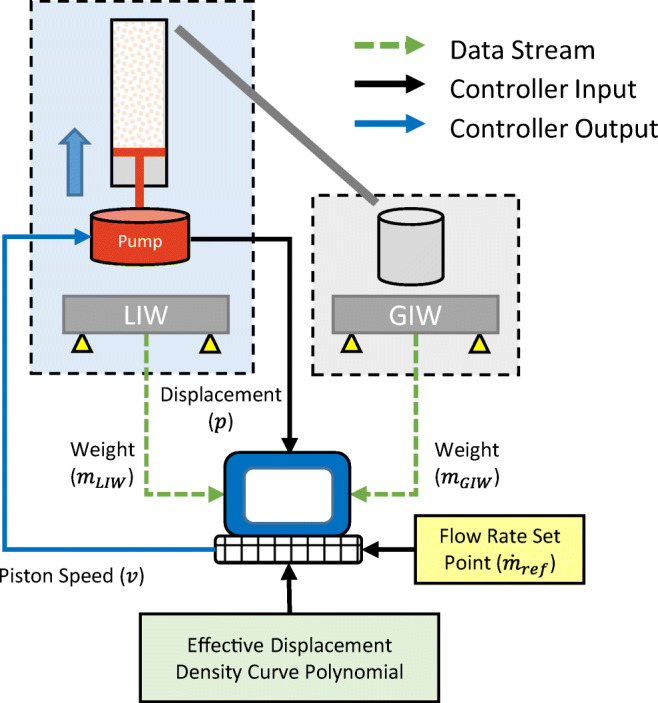
Schematic of the feed-forward control strategy

A schematic of the control strategy is shown in Fig. 3.

#### Iterative Learning Control

An iterative learning control (28, 29) algorithm was developed and implemented in the process controller for correcting an offset in the displacement feed factor profile. Factors that change the effective displacement feed factor (e.g., batch-to-batch variability, inconsistent pre-conditioning, and operator dependency) can result in an offset of the displacement feed factor.

During these runs, the pump was run in the feed-forward control mode based on the displacement feed factor data from the calibration. The LIW data were then used to compensate for disturbances at regular time intervals. Specifically, a deviation from the dosed mass obtained from the LIW data was compared to the desired dosed mass according to the set-point. This error was used to correct the offset coefficient, *α*_0_, in Eq. (3). However, the offset only affected the last polynomial coefficient and did not alter the shape of the displacement feed factor curve. This offset correction was applied after a certain predefined time. We call this method the “corridor control” principle. Alternatively, it can be applied at a certain piston displacement. After the time interval or displacement that corresponded to one iteration, the actual measured feed rate was once again compared to the set-point, and the polynomial function was updated accordingly. In our case, coefficient *α*_0_ of the polynomial was updated. The update law is given by Eq. (6):

6 $$ {\alpha}_{0,k+1}={\alpha}_{0,k}+K\cdot {e}_k $$

Error *e*_*k*_ is the difference between the desired dosed mass per time (feed rate ${\dot{m}}_{nom}$) and the actual dosed mass per time (feed rate ${\dot{m}}_{LIW}$) during the previous time interval (or displacement interval), *k*. The appropriate choice of constant *K* will be outlined below. The nominal feed rate was assumed to be:

7 $$ {\dot{m}}_{nom}=\left(\sum \limits_{i=1}^8{\alpha}_i{p}^i+{\alpha}_{0,k}\right)\cdot v\cdot {A}_{cart} $$

The actual feed rate is composed of the nominal feed rate and additive disturbance ${\dot{m}}_d$. Under the assumption that only *α*_0_ is uncertain (unknown offset *d*), the actual feed rate can be written as:

8 $$ {\dot{m}}_{LIW}={\dot{m}}_{nom}+\dot{m_d}=\left(\sum \limits_{i=1}^8{\alpha}_i{p}^i+{\alpha}_{0,k}+d\right)\cdot v\cdot {A}_{cart} $$

This actual feed rate should now correspond to the updated polynomial:

9 $$ {\alpha}_{0,k+1}={\alpha}_{0,k}+d $$

The integral deviation of the measured feed rate from the set-point during one integration period, which is equal to one iteration, can be calculated using Eq. (10):

10 $$ {e}_k={\int}_{t-{t}_{int}}^te\left(\tau \right)\  d\tau ={\int}_{t-{t}_{int}}^t\left({\dot{m}}_{nom}-{\dot{m}}_{LIW}\right) d\tau ={\int}_{t-{t}_{int}}^t\left(-d\cdot v\cdot {A}_{cart}\right) d\tau $$

Since *d* and *A*_*cart*_ are constant, after performing the integration of velocity and considering Eqs. (6) and (9), *K* can be computed as follows:

11 $$ {e}_k=-d\cdot {A}_{cart}\ {\int}_{t-{t}_{int}}^tv\  d\tau =-d\cdot {A}_{cart}\ \left[p(t)-p\left(t-{t}_{int}\right)\right] $$

12 $$ K=\frac{d}{e_k}=-\frac{1}{A_{cart}\ \left[p(t)-p\left(t-{t}_{int}\right)\right]} $$

Furthermore, for a time-invariant set-point ${\dot{m}}_{set}$, Eq. (10) can be simplified to Eq. (13) for the benefit of using the measured mass values from the balance, which provides inherently integrated values of the feed rate:

13 $$ {e}_k={\int}_{t-{t}_{int}}^t\left({\dot{m}}_{nom}-{\dot{m}}_{LI W}\right) d\tau ={\dot{m}}_{nom}\cdot {t}_{int}+\left({m_{LI W}}_t-{m}_{LI{W}_{t-{t}_{int}}}\right) $$

**Fig. 4:**
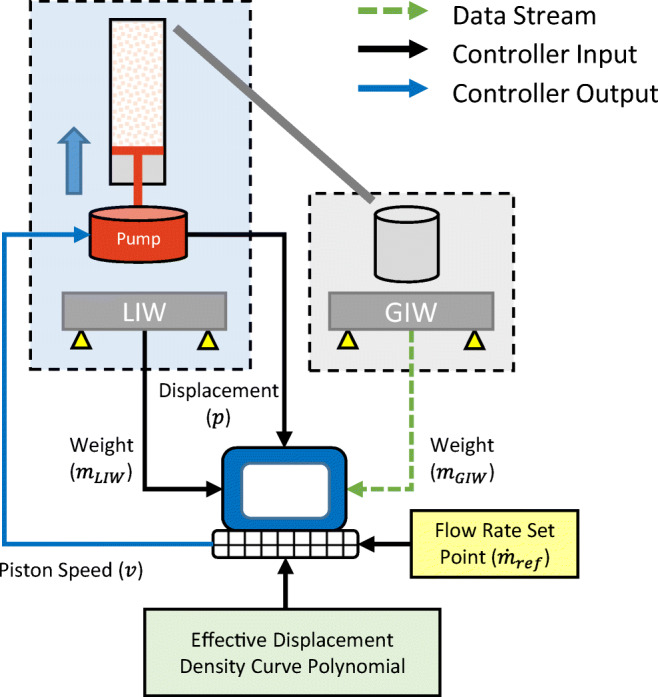
Schematic of the iterative learning control strategy

A schematic of the control strategy is shown in Fig. 4.

To show the effect of iterative learning control, the initially well-fitted polynomial displacement feed factor profile used in feed-forward control was offset by − 10%, resulting in a 10% higher displacement speed compared to the set-point at the beginning of the experiment. Start-up and integration time were chosen in accordance with a filtering window time.

The algorithm was then used to control the actual micro-feeding system, and two experiments were performed. First, the iterative learning control based on the LIW balance data was tested. Second, a feed-forward controlled run using the wrong polynomial displacement feed factor was run for the purpose of quantifying the capabilities of iterative learning control. During this run, the displacement speed was the one obtained in the calibration run but increased by 10% according to the disturbance introduced. No control action based on LIW balance data was taken.

#### Feeding Performance Metrics

The feeding performance evaluation of the feed rate $\dot{m}$ is based on the following standardized methods: the average relative standard deviation (*RSD*_%_) given by Eq. (16), with *s* denoting the standard deviation and the average relative deviation from the set-point (*RDtS*_%_) as a quotient of the average deviation from the set-point over the feed rate set-point${\dot{m}}_{set}$ (see Eq. (18)). The relative deviation of mean to set-point (*RDMtS*_%_) is calculated by Eq. (18), where ${\dot{m}}_{mean}$ is mean feed rate.

14 $$ {\dot{m}}_{mean}=\frac{1}{N}\sum \limits_1^N\dot{m} $$

15 $$ s=\sqrt{\frac{\sum \limits_1^N{\left(\dot{m}-{\dot{m}}_{mean}\right)}^2}{N}} $$

16 $$ RS{D}_{\%}=\frac{s}{{\dot{m}}_{mean}}\cdot 100 $$

17 $$ RD{tS}_{\%}=\frac{\frac{1}{N}\sum \limits_1^N\left|\dot{m}-{\dot{m}}_{set}\right|}{{\dot{m}}_{set}}\cdot 100 $$

18 $$ RDM{tS}_{\%}=\frac{\left|{\dot{m}}_{mean}-{\dot{m}}_{set}\right|}{{\dot{m}}_{set}}\cdot 100 $$

## RESULTS AND DISCUSSION

Di-calcium phosphate, croscarmellose sodium, and barium sulfate were used for evaluating the performance of the feed-forward control strategy. The main reason for choosing these materials was to represent a spanning range of material properties (e.g., the particle size distribution and flow properties). The powder properties of the investigated materials are summarized in Table 1. Di-calcium phosphate is an example of a large-particle system (×50 = 184 μm) with a fair flowability (1.19 < *H*_*R*_ < 1.25), and croscarmellose sodium represents a small-particle system (×50 = 43 μm) with a very poor flowability (1.46 < *H*_*R*_ < 1.59). As our previous study (30) indicated, systems with similar PSDs have qualitatively the same displacement feed factor in the micro-feeder system. Barium sulfate was chosen to represent an extremely small particle system (×50 < 10 μm) with an extremely poor flowability (*H*_*R*_ > 1.6).

**Table 1:** Powder Properties of the Investigated Materials. ± Represents One Standard Deviation (*n* = 3)

Materials	Di-calcium phosphate	Croscarmellose sodium	Barium sulfate
X10 (μm)	14.6 ± 1	19.5 ± 0	0.74 ± 0
X50 (μm)	184.4 ± 5	43.4 ± 0	3.06 ± 0
X90 (μm)	314.5 ± 3	119.5 ± 2	7.94 ± 0
Hausner ratio (***H***_***R***_)*	1.24	1.51	1.81
Bulk density (g/cm^3^)	0.70 ± 0.00	0.51 ± 0.00	0.73 ± 0.00
Tapped density (g/cm^3^)	0.87 ± 0.00	0.77 ± 0.00	1.32 ± 0.00

*H_*R*_*tapped density/bulk density*

### Calibration Runs: Defining the Displacement Feed Factor

**Fig. 5:**
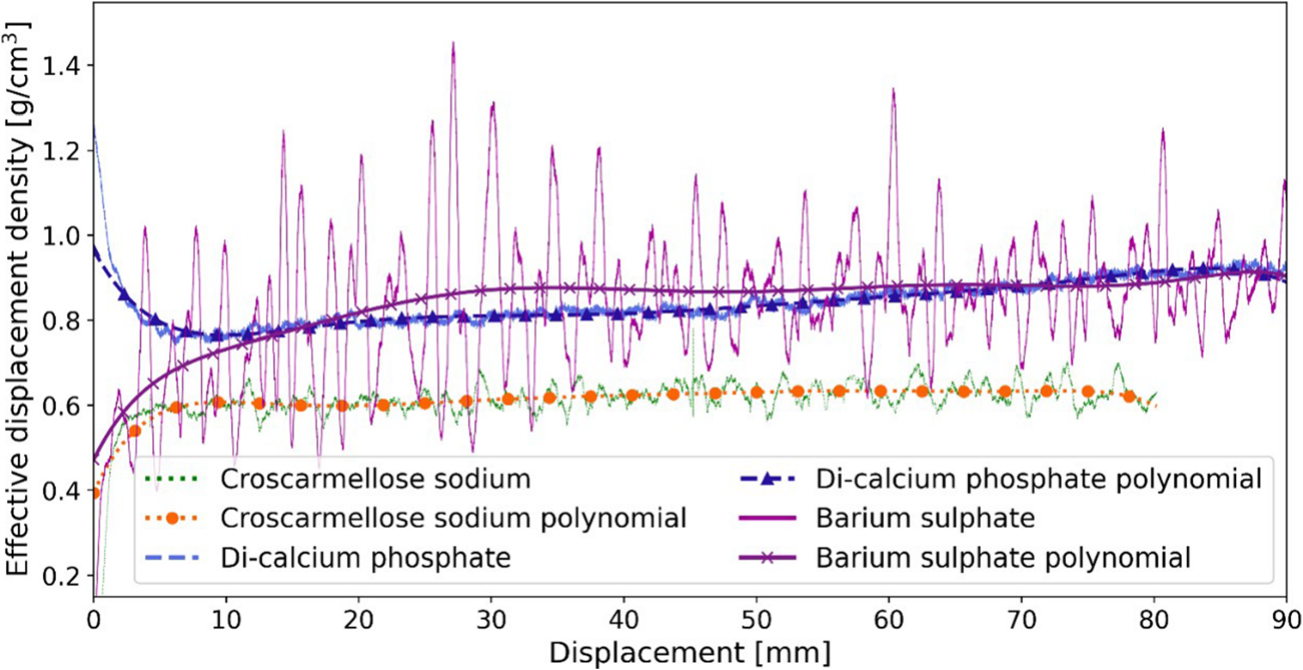
Displacement feed factor profile and the fitted polynomial function for all three materials, calculated using the 10 g/h feed rate calibration run (without control) based on the GIW balance data

In the calibration runs, the piston displacement speed was set to a fixed value for the entire run. The piston displacement speeds in all calibration runs are summarized in Table 2. The results of calibration runs are shown in Fig. 5. These are the displacement feed factor profiles of all three materials investigated. The effective displacement density of each material was calculated using the 10 g/h feed rate calibration run (without control) based on the GIW balance data. Results of the calibration runs demonstrate that the displacement feed factor is unique for each material. For di-calcium phosphate, the effective displacement density is higher in the beginning and later decreases to a minimum value. Afterward, the effective displacement density increases again during the feeding process. Croscarmellose sodium shows a constant increase in the effective displacement density data along the cartridge. Barium sulfate shows strong fluctuations due to its very cohesive nature (see *H*_*R*_ in Table 1). The effective displacement density of barium sulfate is lower in the beginning and becomes denser during the feeding process. Based on these displacement feed factor profiles, the polynomial function (Eq. (3)) can be defined for each material and used for adjusting the piston displacement speed to achieve a constant feed rate.

**Table 2:** Summary of Feed Rates, Average Relative Deviation from the Set-Point, and Average Relative Standard Deviation in the Calibration and Feed-Forward Runs

**Material**		**Barium sulfate**			**Croscarmellose sodium**			**Di-calcium phosphate**		
**Piston displacement speed [mm/min]**	**Cal**	0.45	0.22	0.1	0.61	0.31	0.1	0.47	0.23	0.1
**Evaluation range [mm]**	**–**	5–85	5–40*	5–35*	5–75**	5–75	5–75	5–85	5–40*	5–35*
**Set-point [g/h]**	**Cal**	10	5	2.23	10	5	1.64	10	5	2.15
	**FF**	10	5	2.28	10	5	1.66	10	5	2.41
**Mean feed rate [g/h]**	**Cal**	9.53	3.94	1.92	9.65	4.72	1.52	9.67	4.67	1.94
	**FF**	9.98	4.71	2.15	10.06	4.97	1.57	9.88	5.05	2.25
**Relative deviation of mean to set-point, RDMtS [%]**	**Cal**	4.7	21.2	13.9	3.5	5.6	7.3	3.3	6.6	9.8
	**FF**	0.2	5.8	5.7	0.6	0.6	5.4	1.2	1.0	6.6
**Average relative deviation to set-point, RDtS [%]**	**Cal**	13.7	33.0	30.7	4.6	8.1	9.9	5.3	6.6	10.0
	**FF**	7.2	14.3	26.5	2.8	5.5	7.4	1.3	1.6	6.6
**Average relative standard deviation, RSD [%]**	**Cal**	18.0	45.9	40.8	4.2	9.4	10.0	5.3	1.6	3.7
	**FF**	9.9	17.8	35.5	3.5	6.8	11.1	0.9	1.7	2.3

*Cal calibration run, FF feed-forward control run, RDMtS relative deviation of mean to set-point, RSD relative standard deviation*

*The evaluation range was chosen to compare the control results to the calibration ones (which were kept shorter)

**Steady state range is shorter due to the longer pre-conditioning (22–23 mm compression)

**Fig. 6:**
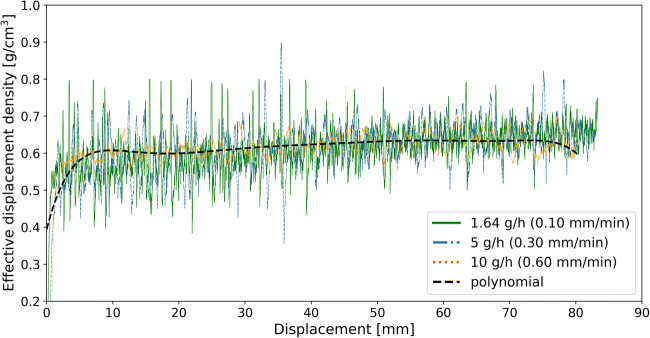
Displacement feed factor profile at all three feed rates selected for croscarmellose sodium; data is obtained from the calibration runs (without control) based on the GIW balance data. The shown polynomial function fitted to the 10 g/h feed rate curve fits to all other feed rates as well. The piston displacement speeds are provided in brackets

As pointed out above, the effective displacement density profile is unique for every material but does not depend on the displacement speed. This is shown in our previous study (30) for other relevant materials, including silicon dioxide with a very small particle size and poor flowability. Figure 6 shows the effective displacement density at three piston displacement speeds for croscarmellose sodium. The shapes and slopes of the curves for different feed rates (piston displacement speeds) are almost identical. Most importantly, the polynomial function fitted to the 10 g/h feed rate curve matches those of the other two feed rates well. Therefore, the polynomial function of the 10 g/h feed rate can be used to model the effective displacement density at all selected feed rates, which can be extended to the two other materials as well.

### Feed-Forward Control

In this section, the ability of the feed-forward control strategy to minimize deviations from the set-point was evaluated. For this purpose, the fitted polynomial function at the 10 g/h feed rate, shown in Fig. 5, was used to predict the effective displacement density. Subsequently, this polynomial function was used to adjust the piston displacement speed in each position to ensure a constant feed rate. Since the displacement feed factors for the investigated materials are not affected by the feed rate (e.g., see Fig. 6 for croscarmellose sodium), the polynomial function fitted to the 10 g/hset-point was used to control the runs at various feed rates for each material. The feed-forward control strategy was evaluated by comparing the control runs to the calibration runs in terms of deviation from the set-points (see Eq. (17) and Eq. (18)) and the RSD (see Eq. (16)).

**Fig. 7:**
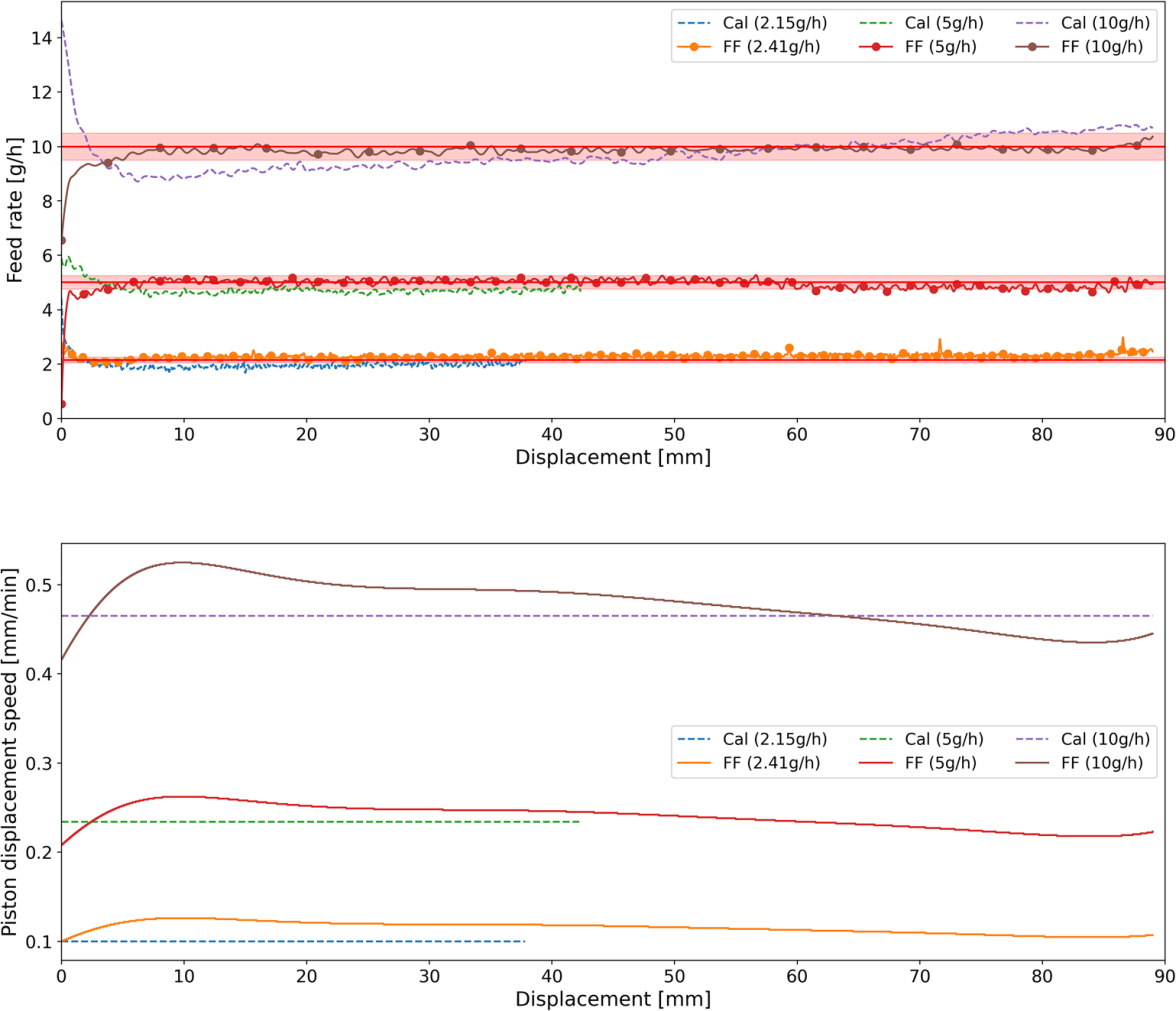
Feeding of di-calcium phosphate at various feed rate set-points: comparison of feeding without (Cal) and with control (FF). The piston displacement speed for the calibration run was set to 0.47 mm/min, 0.23 mm/min, and 0.1 mm/min, respectively, for feed rate set-points of 10 g/h, 5 g/h, and 2.15 g/h. For better visibility, the set-point ± 5% is shown with a highlighted red line

**Fig. 8:**
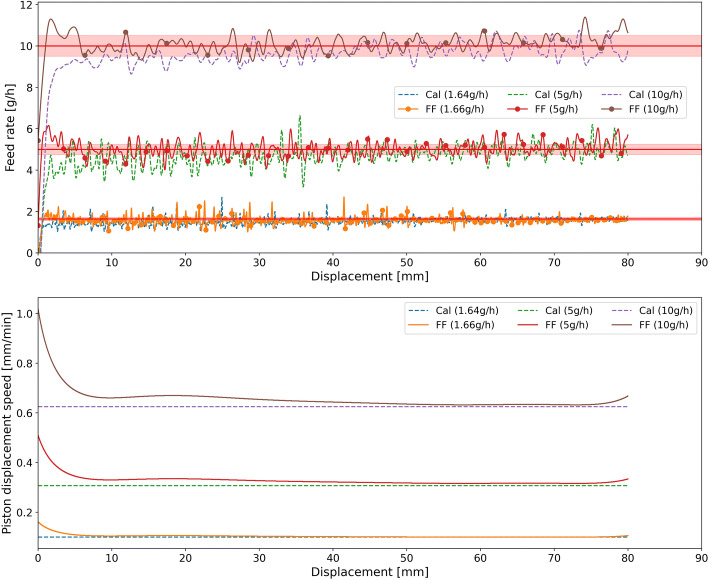
Feeding of croscarmellose sodium at various feed rate set-points: comparison of feeding without (Cal) and with control (FF). The piston displacement speed for the calibration run was set to 0.61 mm/min, 0.31 mm/min, and 0.1 mm/min, respectively, for feed rate set-points of 10 g/h, 5 g/h, and 1.64 g/h. For better visibility, the set-point ± 5% is shown with a highlighted red line

**Fig. 9:**
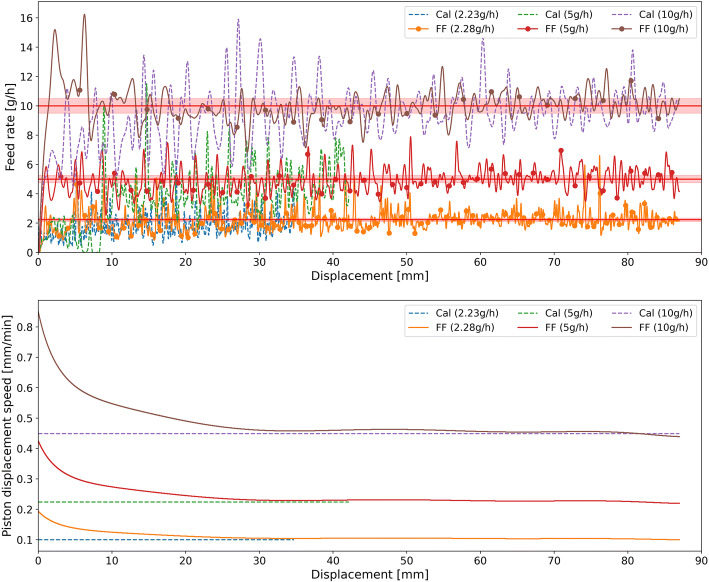
Feeding of barium sulfate at various feed rate set-points: comparison of feeding without (Cal) and with control (FF). The piston displacement speed for the calibration run was set to 0.45 mm/min, 0.22 mm/min, and 0.1 mm/min, respectively, for feed rate set-points of 10 g/h, 5 g/h, and 2.23 g/h. For better visibility, the set-point ± 5% is shown with a highlighted red line

The feeding curves of di-calcium phosphate, croscarmellose sodium, and barium sulfate in the calibration and feed-forward control runs are shown in Figs. 7, 8, and 9, respectively. The figures provide comparisons of the feed rate and the piston displacement speed in the calibration and control runs for all three feed-rate set-points. As Figs. 7, 8, and 9 indicate, the control runs were executed until the cartridge was empty, while the calibration runs were shorter for the lower feed rate set-points of di-calcium phosphate and barium sulfate. This is because the micro-feeder system is very stable over the studied range. As shown in Fig.6 for croscarmellose sodium, the polynomial function fitted to the 10 g/hset-point fits the other set-points as well. Due to this fact, for other materials, the calibration runs for lower set-points (< 10 g/h) were done only for reduced amounts of time. The reason for fitting the polynomial function of 10 g/h feed rate to the other feed rates is that this run is the shortest one in terms of time and the longest one in terms of displacement. Therefore, in a short time, all required information, which is required to control the feed rate of a material, can be obtained. Hence, the duration of calibration runs at various feed rates differs in terms of displacement in Figs. 7 and 9 while the lower set-points were executed with a maximum duration of 6 h. The execution time of the lowest possible feed rate with a piston displacement speed of 0.1 mm/min was 6 h, which is equivalent to 36 mm displacement. The execution time at the 5 g/h feed rate was 3 h (displacement of 60 mm), while the 10 g/h run lasted less than 2 h (displacement of 90 mm).

Figure 7 shows that the feed rate of the calibration runs for di-calcium phosphate is very high in the beginning and subsequently decreases to a minimum value. Afterward, the feed rate increases continuously with a constant slope. The feed rate of the calibration run for 10 g/hset-point is at the desired set-point only for a short period (displacement of 55–70 mm). However, during the feed-forward controlled run, the feed rate is at the set-point for the entire run. A comparison of the piston displacement speed in the calibration runs (set to a fixed value for the entire run) and the control runs (changed based on the model) is provided in Fig. 7.

The same improvement in the feed rate deviation from the set-point can be observed for croscarmellose sodium in Fig. 8. The feed rate of the calibration runs for croscarmellose sodium is out of the desired set-point for the first 40 mm of displacement. However, the control runs show a much closer feed rate to the set-point along the entire run.

The feed rate deviation for barium sulfate improved remarkably in the control run as well. As shown in Fig. 9, the feed rate is much lower than the set-point at the beginning of the calibration run (less than half of the set-point for almost 10 mm for 10 g/h set-point). The reason is that pre-conditioning was performed differently for this material due to setup issues. Barium sulfate was only tapped and not compacted to the tapped density state, which may be the main explanation for the observed low feed rate at the beginning of the calibration run. Nevertheless, the feed-forward control run led to much smaller feed rate deviation from the set-point. The last point was particularly notable at the beginning of the run.

All in all, the results show that, despite pre-conditioning prior, the powder density is not constant or does not remain constant along the cartridge. This leads to feed rate deviation from the set-point when no control is applied (calibration runs). However, what was noted was the changes in density along the cartridge were reproducible and measurable using an effective displacement density profile. Using this profile, the feed-forward controlled runs were able to reach the specified set-point and maintain a stable feed rate at this level. It is important to note that even with the implementation of a feed-forward control, there was a short start-up phase where deviations from the set-point were notable. These deviations were most pronounced for di-calcium phosphate, possibly due to an initially larger deviation between the model and the measurement data of the displacement feed factor.

Results for the feed rate set-points of all materials, deviation from set-points as well as average relative standard deviation in the controlled and calibration runs, are summarized in Table 2. The results were compared for the control and calibration runs by determining the RSD (see Eq. (16)) and the feed rate deviation from the set-point (see Eq. (17) and Eq. (18)). RSD represents the distribution of feed rate measurements around the average feed rate normalized by the average feed rate. The deviation from the set-point is a measure of how close the set-point is met.

Table 2 indicates that the feed rate set-point in the calibration runs and the feed-forward controlled runs is not the same at the lowest feed rate. Since the minimum possible piston displacement speed was 0.1 mm/min (syringe pump limitation), the piston displacement speed was fixed at this value in the calibration runs at the minimum throughput. However, the minimum throughput in the feed-forward control runs is determined by the maximum displacement feed factor in combination with the minimum piston displacement speed. This minimum set-point can be calculated by rearranging Eq. (5) and using the maximum displacement feed factor. Using a lower feed rate set-point would result in a truncation of the piston displacement speed at 0.1 mm/min. This equipment limitation hinders achieving the desired set-point. Therefore, the feed rates at the lowest piston displacement speed cannot be compared to the lowest possible set-point for the feed-forward control, which takes the displacement feed factor into account.

The mean feed rate and RSD data in Table 2 show a significant improvement in the feeding consistency for all materials using the feed-forward control strategy. The deviations from the set-point (RDtS and RDMtS) are significantly lower in all control runs. Notably, the RDMtS (Eq. (18)) is the absolute difference between set-point and measured feed rate; however, the RDtS (Eq. (17)) is considering the average absolute difference, and thus, positive and negative deviations do not compensate each other. Therefore, the RDMtS provides a better estimation, and it is of course lower. The RDMtS decreased to lower than 7% in various set-points of feed-forward control runs.

RSD decreased for all control runs except for 5 g/hset-point of di-calcium phosphate and minimum set-point of croscarmellose sodium. Generally, the RSD is caused by deviation from the set-point, inconsistencies in feed rate and measurement noise (from the scale). As the feed rate is stable (following a horizontal profile in the considered range) for the feed-forward controlled experiments, the effect of this deviation to the set-point is removed, and the remaining RSD can be attributed to material-inherent inconsistencies in feed rate as well as measurement noise. In particular, for croscarmellose sodium, the RSD reduction is lower compared to other materials, since the effective displacement density is less dependent on the displacement (see Fig. 8). Furthermore, a higher RSD at lower throughputs indicates that the RSD’s absolute value is determined by the measurement noise, which is not affected by the throughput.

The RDtS is an important measure to quantify the feeding performance. It is clearly smaller for all feed-forward controlled runs compared to the calibration. A clear trend can be observed: a relative deviation from the set-point is lower at higher feed rates, which—in combination with the RSD—suggests that the feeding performance is better at higher throughputs. The largest deviation from the set-point of 2.28 g/h(cal) versus 2.15 g/h(FF) at the minimum throughput is observed for barium sulfate. This can be translated to feeding 0.13 g less of material in 1 h. The deviation is most likely caused by a non-reproducible displacement feed factor curve due to inconsistencies during filling and pre-conditioning.

In summary, a good feeding consistency was achieved using the feed-forward control strategy. Nevertheless, to compensate for any deviation, an iterative learning control approach was adopted.

### Iterative Learning Control

The concept of iterative learning control is demonstrated by introducing an error (artificial offset) in the polynomial density model. This error, mimicking a 10% lower density than the one obtained in the calibration runs, results in a higher displacement speed and thus a too high feed rate. The iterative learning approach was tested as a proof of concept using the micro-feeding system for di-calcium phosphate at a feed rate set-point of 10 g/h. The iterative learning control (iterative learning/feed-forward combined) was then compared to having only feed-forward control in place, with the same 10% higher feed rate. The basic idea of this concept is that LIW control, as it is used by standard LIW feeders, does not work well for the micro-feeder, since the mass loss over time is low, and scale resolution is usually not good enough to provide data for the feed-back control in sufficiently small time intervals. Thus, we propose a concept where control is mostly feed-forward, and weight-loss data are used for an iterative learning approach. The iterative learning approach assures that the feed-forward controlled feed rate remains within the permissible range of feed rates in order to achieve products with CQAs meeting approved targets. We call this concept a “corridor-control” approach. In this case, we demonstrate the corridor approach with a feed rate of 10 g/h with a corridor of ±7.5%. This corridor is chosen considering the accuracy and readability of GIW (0.0001 g) and LIW (0.1 g) balances.

In general, the iterative learning control consists of the initialization phase and the iteration phase. The initialization phase is used to reach a stable feed rate. During this phase, the controller is not active. During the iteration phase, the controller is active and tracks the reference (set-point) in an iterative manner, either on iteration displacement or iteration time interval (applied in this work).

In the micro-feeder system, the LIW balance data are used for iterative learning control. An initialization step of 600 s was chosen. These 600 s (10 min) are chosen based on the displacement speed of approximately 0.5 mm/min at the beginning of the experiment, corresponding to a piston displacement of 5 mm. It can be seen in Fig. 7 that after 5 mm at a feed rate of 10 g/h, a steady state is reached. Subsequently, the iteration step with an integration period of 1200 s was applied. This means that every 1200 s, the polynomial density model is updated to keep the feed rate close to the desired feed rate set-point.

The weight loss recorded by the LIW balance is used to calculate the correction term and update the polynomial for the next iteration step. The LIW balance values are smoothed by using a linear polynomial fit over the 60 s before the iteration step starts. This is done to obtain more robust results from the LIW balance, where disturbances, especially introduced by the scraper, influence the LIW balance data. The error for the polynomial density model is calculated from Eq. (13). This approach is done in a repetitive mode for each iterative step.

**Fig. 10:**
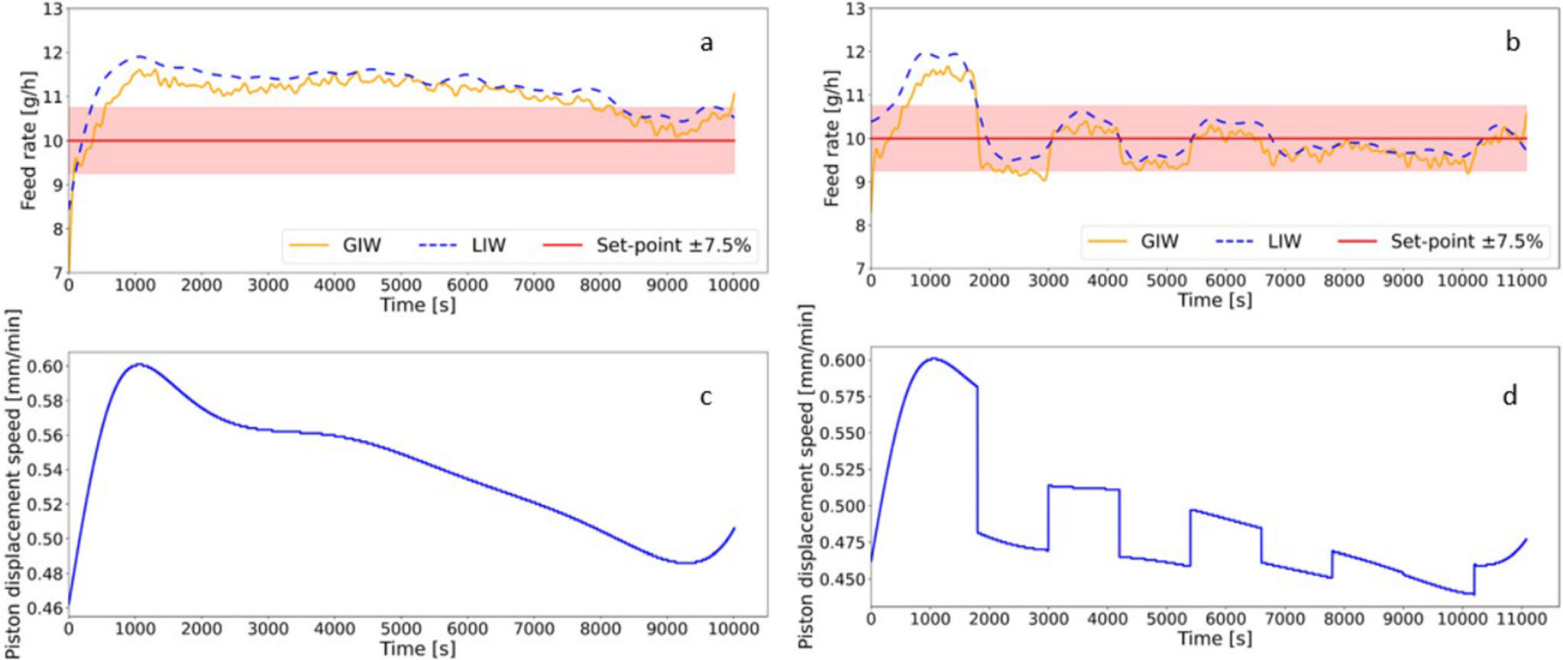
Comparison of iterative learning (IL) control active (**b**, **d**) to IL inactive (**a**, **c**) runs: di-calcium phosphate with a 10% feed rate off set. **a**, **b** The feed rate as a function of time for IL control inactive and IL control active runs, respectively. Feed rates generated from both LIW scale (blue dotted line) and GIW scale (yellow line) data are presented. For better visibility, the set-point of 10 g/h ± 7.5% is shown with a highlighted red line. **c**, **d** The displacement speed profile of the syringe pump as a function of time for, respectively, IL control inactive and IL control active runs. The LIW scale signal is used for adapting the piston displacement speed

Figure 10 shows a comparison of iterative learning control active (corridor-control, Fig. 10b) and only feed-forward control (Fig. 10a). The results are summarized in Table 3. As mentioned, Fig. 10a depicts the feeding of di-calcium phosphate with a 10% offset error in the initial feed rate. In the beginning, the feed rate is increasing and reaching a (too high) steady state after an initialization period. Towards the end of the experiment, the feed rate is decreasing. This can be explained by a deviation from the effective displacement density compared to the calibration experiment for which the polynomial density model was obtained to adjust the piston displacement speed. Possibly, pre-conditioning was not as effective in this illustrative example. As can be seen in the figure, the feed rate is out of spec for almost the entire run, since the control variable adjustment is not error-based in the feed-forward control concept. In fact, two contributions to the off-specification feeding performance can be identified: first, a 10% offset, and second, a decrease of density during the run in contrast to the calibration experiments.

**Table 3:** Summary of Iterative Learning Control (IL) Active and Inactive Feeding Results Based on GIW and LIW Scale Data. Material Fed: Di-Calcium Phosphate

**Scale for Data**	**Set-point (g/h)**		**Mean feed rate (g/h)**		**Relative deviation of mean to set-point, RDMtS (%)**		**Average relative deviation to set-point, RDtS (%)**		**Average relative standard deviation, RSD (%)**	
	**IL off**	**IL on**	**IL off**	**IL on**	**IL off**	**IL on**	**IL off**	**IL on**	**IL off**	**IL on**
**GIW**	10	10	11.08	9.90	10.8	1.0	10.76	5.10	3.12	6.45
**LIW**	10	10	11.31	10.14	13.1	1.4	13.11	4.98	3.19	6.83

*GIW gain-in-weight, LIW loss-in-weight, IL iterative learning*

Figure 10b shows the corridor-control (feed-forward/iterative learning control combined) feeding of di-calcium phosphate with a 10% error. Compared to Fig. 10a, there are stepwise changes in the feed rate apparent. At the initialization step (600 s) and the first iteration step (integration period of 1200 s), the feed-forward control was active. Therefore, the feed rates for the first 1800 s in Fig. 10a, b are similar. However, after 1800 s, the computation of the difference between reference (set-point) and measured dosed mass (LIW balance signal recorded during the first iterative step) leads to a set-point offset, and the controller reduces the displacement speed. In the next iteration, at 3000 s, the feed rate is too low, and therefore, the controller increases the displacement speed again. However, the controller is still following the initial shape of the polynomial density model for adjusting the displacement speed and is only adding or cutting the error. This approach keeps the feed rate close to the set-point in the defined corridor.

There are slight differences in the LIW and GIW data, which is due to the difference in accuracy and readability of these balances. Since the correction in the corridor control approach is based on the LIW balance data, only considering the LIW data, the feed rate is after the first iterative step for the entire run in the range of the set-point ±5%. From a processing and GMP perspective, the LIW balance information can be used to provide material accountability at the end of the run and enable tracking of material in real-time over a longer corridor. It is understood that the precision of the LIW balance will not be sufficient to control the process over short process windows where only milligrams (mg) of material are dispensed; however, over longer periods of time, the data can be beneficial.

Introducing stepwise changes in the displacement speed profile and hence in the feed rate increases the RSD of the signal. However, as can be seen from Table 3, the RDMtS and RDtS are drastically reduced by means of iterative learning control. The RDMtS is reduced to less than 1.5% in iterative learning active runs. The RDtS is considering the average absolute difference between set-point and measured feed rate so the positive and negative deviations do not compensate each other. Therefore, this example shows that the RSD is not a suitable single measure for evaluating the feeding performance.

For the corridor control approach (iterative learning control active), the displacement speed decreases to a much lower speed after the first iteration step (see Fig. 10d at 1800 s). This leads to a sharp decrease in feed rate and therefore resulting in a high RSD. Refinements on the timing of initialization step and iteration step are currently under investigation. Moreover, a reduction of the iteration step duration will allow a faster reaction to certain process disturbances however on the cost of robustness. Therefore, variable timing based on noise level is under consideration.

## SUMMARY AND CONCLUSIONS

A two-stage control strategy was developed for a novel micro-feeder system. The performance of the feed-forward control strategy was evaluated using di-calcium phosphate, croscarmellose sodium, and barium sulfate representing powders with different material properties and feeding characteristics. A material-specific displacement feed factor, which is not affected by the feed rate, was obtained in the calibration runs for each material. This factor was used to predict the feed rate and proactively control the piston displacement speed as a manipulated variable. The influence of effective displacement density variation on the feed rate during processing was successfully minimized. Stable feed rates were achieved at the set-point levels via pre-defined modification of the piston displacement speed based on the prediction model (obtained in the calibration runs). The relative deviation from the set-point and the RSD decreased significantly, particularly for the materials with high feed rate fluctuations without control.

Furthermore, an iterative learning control strategy combined with the feed-forward control was developed and successfully transferred to the physical micro-feeding system. As a proof of concept, di-calcium phosphate was fed at 10 g/h using this control strategy to demonstrate its applicability to the micro-feeder. The results are highly promising, and further optimization and refinements will be carried out in a follow-up study.

The results of this study attest to the potential of the proposed micro-feeder system for industrial implementation. By applying the proposed control strategy, the feeding performance of materials that are difficult to handle at low doses using conventional systems can be improved to fulfill the requirements of commercial manufacturing.
